# The Antiviral Properties of Human Milk: A Multitude of Defence Tools from Mother Nature

**DOI:** 10.3390/nu13020694

**Published:** 2021-02-22

**Authors:** Daniela Morniroli, Alessandra Consales, Beatrice Letizia Crippa, Giulia Vizzari, Federica Ceroni, Jacopo Cerasani, Lorenzo Colombo, Fabio Mosca, Maria Lorella Giannì

**Affiliations:** 1 Department of Clinical Sciences and Community Health, University of Milan, 20122 Milan, Italy; daniela.morniroli@unimi.it (D.M.); giulia.vizzari@unimi.it (G.V.); federica.ceroni@unimi.it (F.C.); jacopo.cerasani@unimi.it (J.C.); fabio.mosca@unimi.it (F.M.); maria.gianni@unimi.it (M.L.G.); 2 Fondazione IRCCS Cà Granda Ospedale Maggiore Policlinico, Neonatal Intensive Care Unit, 20122 Milan, Italy; beatriceletizia.crippa@gmail.com (B.L.C.); lorenzo.colombo@mangiagalli.it (L.C.)

**Keywords:** human milk, viruses, SARS-CoV-2, lactoferrin, tenascin-C, immunoglobulins, docosahexaenoic acid (DHA), long-chain polyunsaturated fatty acids (LCPUFA), mucins, human milk oligosaccharides (HMO)

## Abstract

The anti-infective properties of breast milk have been known for decades. In recent years, an increasing number of papers have described the variety of bioactive compounds that are present in breast milk with varying degrees of antiviral activity. However, to date, the totality of the properties of these compounds is not fully understood and, above all, their synergistic interaction is not yet known. The purpose of this review is to describe the current knowledge about the antiviral compounds in breast milk, both with specific and non-specific action against pathogens. Due to the current pandemic situation from SARS-CoV-2 (Severe acute respiratory syndrome Coronavirus-2), research has focused on a multitude of potential antiviral substances, taking breast milk as a biological model of reference. Future research is needed to expand the knowledge of these compounds, which will hopefully assist in the development of therapies applicable even at later ages.

## 1. Introduction

Breast milk is now known as an incomparable source of bioactive substances that contribute to the newborn’s development and health. In recent years, there has been increased interest in human milk’s numerous compounds with promising immunological and antimicrobial effects [1]. It is now well known that breast milk has an overall protective effect on the newborn, particularly regarding the development of immunity and protection from infections [2].

The first systematic review describing the link between breastfeeding and the protection of newborns from infectious diseases was published in 1935 [3]. Since then, a growing amount of literature has demonstrated that the breastfed newborn is at lower risk of having a gastrointestinal viral disease or respiratory infection [4,5,6,7]. In addition, human milk feeding was correlated with protection against urinary tract infections, ear infections and many other diseases [8]. However, despite the identification of various compounds with antimicrobial effects, research has failed to fully understand human milk’s protective effect. This may be due not only to the presence of innumerable substances, but above all to a still lacking understanding of the hypothesized interactions between these substances and pathogens, such as for human milk oligosaccharides (HMOs) [9] and glycoproteins [10].

At birth, the newborn has not yet reached full immunological competency due to the adaptive immunity components’ immaturity. This immaturity derives from limited exposure to antigens in utero and impaired functions of B and T cells [11]. Therefore, the protection of the neonate against infections mainly depends upon the passively acquired antibodies transferred from the mother, bioactive antimicrobial compounds and the innate immune system [11]. To counteract this susceptibility, breast milk has evolved to concentrate on immunomodulating substances, antibodies and molecules with a direct antibacterial and antiviral function. This protective function is so marked that it has been speculated that the mammary gland could have ontogenically originated from the immune system. This would account for the functional aspect of the colostrum, which has an immunological function even before a nutritional one.

A recent review by Vorbach et al. has speculated about the innate immunity origin of the mammary gland by observing that many of the molecules found in breast milk also have immunological properties (Table 1). The authors suggest that milk could have evolved from a mucus secretion containing many evolutionarily conserved, protective molecules [12]. The combination of these mechanisms is so powerful that early exclusive breastfeeding may reduce the risk of postnatal human immunodeficiency virus infection 1 (HIV-1) transmission from HIV-positive mothers in ongoing treatment with antiretroviral therapy [13,14]. Recently, it has also been seen that the passage of Cytomegalovirus (CMV) through breast milk is strongly inhibited by some substances in the mother’s milk itself, in fact hindering the vertical transmission of the virus [15].

Thanks to the current pandemic situation caused by SARS-CoV-2 (Severe acute respiratory syndrome Coronavirus-2), breast milk’s antiviral properties have returned to be under the spotlight for many researchers worldwide. However, the complex interaction between human milk and viruses is yet to be understood. Recent studies have demonstrated the importance of the viral microbiota (virome) to the infant’s health [16]. In addition to the child’s intestinal bacterial colonization, there is a population of bacteriophages that modulate and regulate bacterial growth [17]. This virome has a dual origin and a sequential course. Liang et al. (2020) investigated how the newborn is first colonized mainly by bacteriophages, which are induced by the first bacteria constituting the microbiota. Subsequently, at around four months of life, human viruses with intracellular replication are also found in the infant’s intestine. Therefore, the quantity and pathogenicity of these human intracellular viruses are reduced if the infant is breastfed [18].

Emerging evidence shows that human milk viruses are also transmitted from the mother to the infant via breastfeeding [16]. The human milk virome has been only briefly investigated, but researchers are beginning to demonstrate a vertical transmission of bacteriophage, suggesting that the mammary gland provides both the bacterial and viral parts of the microbiome [19]. Therefore, it could be speculated that breast milk favors the survival and transmission of viral particles favorable to the neonate’s health and, at the same time, hinders the transmission and replication of pathogenic viral strains.

The purpose of this narrative review is to provide a brief description of the known antiviral compounds in human milk.

## 2. Specific Antiviral Properties

The direct antimicrobial action of breast milk is expressed through all kinds of immunoglobulin (sIgA, IgA, IgG, IgM, IgE, IgD) compounds, with anti-infective activity already known for many decades [8]. The IgG and IgM antibodies derive from the maternal immune response. The variety of viruses that are circulating in a given population influences the pool of memory antibodies present in breast milk—to the newborn’s advantage—as they will therefore be passively immunized to survive in that environment. This protective mechanism allows the newborn to have direct access to a ready-made pool of antibodies that are directed against the infectious agents recognized by the maternal body and that, in all probability, will also come into contact with the child [20]. It has recently been described that the breastmilk of women infected with SARS-CoV-2 could be rich in antibodies directed against this virus, as indeed happens for any other infection [21].

Interestingly, it has also been suggested that there is a retrograde pathway from the newborn to the mother. The microbial agents present in the infant’s mouth, due to a retrograde flux of milk, would make contact with the mammary gland, triggering an immunological response and, therefore, the production of antibodies. These antibodies would then protect the newborn from the microbes it comes into contact with, exploiting the mother’s immune system and supporting the child’s immature one [22].

However, this type of “immunoglobulin therapy” presupposes a past or ongoing maternal viral disease or encounter and is specifically directed towards a given virus.

Breast milk is also rich in secretory IgA (sIgA), with a concentration 10–100-fold higher than that in serum. This antibody has a dual anti-infective action, providing protection from various microbes and modulating intestinal immunity. The abundance of milk sIgA has long been known for its ability to coat the newborn’s gastrointestinal tract, hindering the binding of some specific microbial agents [23]. These monoclonal antibodies are produced in the maternal body, mainly from T-dependent B lymphocytes. They are directed against specific microbial antigens, thus conferring a certain degree of protection against pathogens [24]. Their role in modulating the microbiota is perhaps less well known. Hand et al. (2019) demonstrated that a mother’s breast milk derived IgAs bound with bacteria and hindered their overgrowth in the newborn’s intestine. The authors described how the lack of IgA-bacteria binding can lead to anaerobic bacterial overgrowth and, thus, promote inflammatory diseases such as necrotizing enterocolitis. Although it is not a direct antiviral effect, there is evidence of the importance of breast milk in modulating the newborn’s immunity and intestinal inflammation [25].

## 3. Aspecific Antiviral Properties: The Bioactive Compounds

Among the breast milk compounds with a known broad antimicrobial activity, we also find cytokines, polyunsaturated fatty acids, immune-stimulating proteins, glycoproteins such as lactoferrin, glycated components such as mucins, human milk oligosaccharides (HMOs) and extracellular vesicles in human milk [4]. This wide variety of substances is fascinating because they provide the mother’s milk with a broad spectrum of antiviral properties [4]. Greater knowledge of these bioactive compounds could help identify new strategies to fight viral infections, even at a later age.

A recent study by Donalisio et al. (2020) investigated the antiviral activity of extracellular vesicles (EVs) extracted from the colostrum of mothers who gave birth prematurely. This study demonstrated an antiviral activity of colostrum against CMV and an even more marked activity of the EVs in it, especially concerning some proteins present on their surface. This is particularly interesting when considering the risk of vertical transmission of CMV via breast milk in a susceptible population, such as in premature babies [26].

Cytokines are small proteins with intercellular signaling functions. In breast milk, there is an excellent variety of cytokines, both with anti-inflammatory and pro-inflammatory actions. The Transforming Growth Factor-β (TGF-β) seems to have a marked immunomodulatory and antiviral action among the inflammatory cytokines. TGF-β positively regulates the production of IgA and, therefore, passive immunity. Furthermore, some studies have shown that if the mother is exposed to a high number of microbial stimuli, the mother’s milk has higher levels of TGF-β, thus suggesting there is regulation to benefit the protection of the newborn [27].

Regarding lipids, it was observed 30 years ago that the lipids in breast milk, or rather the products of their digestion, could have a specific antibacterial and antiviral action [28]. More recent studies have confirmed the antiviral activity of fatty acids in breast milk. The lipids in breast milk are collected in large vesicles consisting of triacylglycerols surrounded by a phospholipid membrane. The action of the newborn’s salivary and gastric lipases is to break down the triacylglycerols into monoglycerides and free fatty acids. Free fatty acids, depending on their length, the degree of saturation and the presence of active radicals, exert a marked antibacterial and antiviral activity [29]. In particular, long-chain polyunsaturated fatty acids appear to be those with the most significant antimicrobial activity, both against bacteria and viruses. In fact, in response to an infection, the cells can release a greater quantity of these fatty acids, particularly docosahexaenoic acid (DHA), arachidonic acid (ALA), and eicosapentaenoic acid (EPA). As in the cells, the fatty acids released by gastric lipases modulate lipoxins, resolvins, protectins and maresins, with an intense immunomodulating and antimicrobial action [30]. As with most of the molecules mentioned so far, polyunsaturated fatty acids and their metabolites (prostaglandins, thromboxanes and leukotrienes) have also been studied for their potential action against SARS-CoV-2.

In particular, given their action against enveloped viruses and in regulating inflammation, it has been assumed that their deficiency could worsen the course of the Coronavirus disease that is responsible for the current pandemic. To date, only a few studies have focused on the correlation between the quantity of polyunsaturated fatty acids in breast milk and its antibacterial and antiviral activity [31,32]. Among lipids, the derivatives from the oxidation of cholesterol have been studied for their antiviral activity. Civra et al. have shown that oxysterols, derived from the oxidation of cholesterol and present in breast milk throughout lactation, can inhibit some widespread viruses in the pediatric population (rhinovirus and rotavirus). Notably, among the oxysterols, 25-hydroxycholesterol (25OHC) and 27-hydroxycholesterol (27OHC) seem to have a broader and more marked antimicrobial activity. The concentration of the latter seems to be exceptionally high in the colostrum. It can be considered an additional antiviral compound for all intents and purposes, which is useful for supporting the newborn’s immature immune system [33].

Amongst the functional proteins of breast milk, lactoferrin has recently risen to the headlines for its supposed effect against SARS-CoV-2, as investigated by a large number of studies that were published in the last year [34,35,36,37,38]. Human milk lactoferrin has always been known for its positive regulatory function in iron absorption and its antimicrobial role. This protein can bind to the iron in milk and remove it from bacterial metabolism, preventing the over-growth of pathogens [37]. Lactoferrin also has significant antiviral activity against a broad spectrum of DNA and RNA viruses (i.e., herpes simplex virus, human papillomavirus, human immunodeficiency virus and rotavirus) by inhibiting the viral entry into host cells [38]. This antiviral effect is carried out in two ways. Firstly, lactoferrin can directly read the virus, thus preventing it from adhering to the cell. Secondly, lactoferrin can occupy the binding sites of heparan-sulfate molecules, which are found both bound to the cell membrane and in the extracellular matrix. Many viruses use these as attachments and concentration sites to facilitate binding to the cell receptor [38].

Another breast milk protein with an interesting antiviral activity is Tenascin-C. This large extracellular multimeric protein can inhibit HIV infection by interacting with its envelope domain, effectively neutralizing the retrovirus and inhibiting transmission via breast milk [39]. The potency of this inhibition appears to be significantly higher than that exerted by lactoferrin and is quite similar to that of HIV-1—neutralizing the monoclonal antibody [40]. Tenascin-C’s antiviral effect is also mediated by the interaction between its glycan structure and HIV’s envelope [39]. Therefore, it could be speculated that the same mechanism of glycan interaction may hinder the transmission of other enveloped viruses such as the influenza virus.

Mucins are large glycosylated molecules that are found in a variety of biological fluids. Their particular structure confers viscosity to body fluids, which increases the greater their presence. Among the various mucin subtypes, Type 1 and Type 4 (MUC1 and MUC4) are found in breast milk. These subtypes have demonstrated antiviral activity against HIV, influenza virus and other viruses in vitro [41]. Like the heparan-sulfate molecules described above, their action is based on many sialylated residues that are very similar to those of the cell membrane and that bind to the virus and trap it, preventing it from reaching the cell surface. Considering the cellular targets of SARS-CoV-2, the protective role of mucins has also been assumed against this virus [42]. It has also been speculated that the mucin content of breast milk and its viscosity can increase in the case of maternal infection by some viruses, such as the Zika virus, giving additional protection to the newborn [43]. The same mechanism has been suggested for SARS-CoV-2 [44,45].

Human milk oligosaccharides (HMO) are several unconjugated carbohydrates that have a dual function for the immunity of the newborn. HMOs favor the development of a favorable microbiota with essential repercussions for immunity [46]. It is now known that these molecules are essential prebiotics that nourish the neonatal microbiota, promote the development of bacterial strains that are favorable to health and, thus, carry out an indirect immunomodulating action. In recent years, however, their direct antiviral role has also been demonstrated. HMOs can hinder the binding of some viruses to the host cell, including the norovirus, HIV, influenza virus and rotavirus [9]. Having a glucosidic structure that is similar to that found on the mucosal surface, HMOs act as their soluble decoys, binding the virus to itself and preventing its entry into the cell. This could be why some studies have shown that the rotavirus vaccine appeared to give a reduced antibody response if the infant who received it was breastfed [47]. At the same time, however, a study by Ramani et al. has instead highlighted how the administration of some HMOs could increase the antibody response to vaccination with the rotavirus strain belonging to the P [11] VP4 genotype (Rotavac ^®^ Bharat Biotec, Telagana, India) currently being used in some countries [48].

## 4. Conclusions

The current pandemic situation has renewed interest in compounds with known or supposed antiviral activity. As is often the case for other topics, breast milk is used as a biological model to be studied to extract its secrets, which have been obtained over hundreds and thousands of years. This narrative review cannot be defined as complete and conclusive. Despite the fact that the bioactive compounds of breast milk have been the subject of research for many years now, we still cannot say that we fully understand all their properties or, moreover, their peculiar interactions resulting in human milk having outstanding properties. This discussion aims to underline the importance of breast milk as a functional food for the infant and to be a stimulus for future research projects aimed at isolating and exploring the components of the most polyvalent compound—breast milk.

## Figures and Tables

**Table 1:** The main groups of antimicrobial compounds in human milk.

**Immunity Cells**	Macrophages 60%, Neutrophils 25%, Lymphocytes 10%
**Antiviral Specific Compounds**	
**Adaptive Immunity Compounds**	Immunoglobulins sIgA, IgA, IgG, IgM, IgE, IgD
**Antiviral Nonspecific Compounds**	
**Cytokines**	IL-1b, IL-2, IL-4, IL-5, IL-6, IL-8, IL-12, IL-13, IL-16, IL-18, IL-10, IFNg, TNFa, G-CSF, M-CSF, GM-CSF, TGFb1 and -2, sCD14
**Innate Immunity Agents**	Complement, Chemotactic Factors, Interferon, α-Fetoprotein, Mannose Binding Lectin, β-Defensin-1 Antiadherence substances: Oligosaccharides, Mucins, Lactadherin, Glycolipids and Glycosaminoglycans, K-Casein Milk Fat Globule Antiviral Factors: Fatty acids and Monoglycerides
**Carrier Proteins and Enzymes**	Lactoferrin, Transferrin, Vitamin B-12 binding protein, Steroid binding protein, Leukocyte enzymes, Antiproteases, Platelet-Activating-Factor, Nucleotides, Long-Chain Polyunsaturated Fatty Acids

IL: Interleukin; IFN: Interferon; TNF: Tumor-necrosis factor; G-CSF: Granulocyte colony-stimulating factor; M-CSF: macrophage colony-stimulating factor; GM-CSF: Granulocyte-macrophage colony-stimulating factor; TGF: Transforming growth factor; sCD14: soluble cluster of differentiation 14.

## Data Availability

Not applicable.
